# Book Reviews

**Published:** 1821-10

**Authors:** 


### CRITICAL ANALYSES

OF

### RECENT PUBLICATIONS, IN THE DIFFERENT BRANCHES OF MEDICINE AND SURGERY,

### In the Literature of Foreign Nations.

---

Πατρίδες ἄρα
Ἀνδράσιν, ὦ πατραῖς ἄνδρες, ἀγαλλόμεθα.

---

*Examen des Doctrines Medicales, &c.; or, an Inquiry into the various Medical Doctrines and Systems of Nosology of the Day; embracing an Account of the System of Medicine now generally adopted, and preceded by an aphoristical Statement of Physiological Medicine.* By F. I. V. Broussais, Principal Physician and Professor in the Military Hospital of Instruction of Paris, &c. &c. &c.

THE French can no more live without a downright, well-concocted system of medicine, with its several apophthegms, propositions, and *laws*, than they can exist, since they have learned politically to think for themselves, without a charter or a constitution. The latter have not been subjected to more changes and alterations than the former have experienced; and Dr. Broussais' book affords another example of the propensity of our spirited and ingenious neighbours to legislate in physic, with that same facility which, of late years, they have shown in framing laws and constitutional charters for themselves and other nations in Europe. But, although their political constitutions may have benefited the condition of the people, it is certain that their medical systems, or *charters*, have seldom improved the people's constitutions. We do not exclude from this general assertion the "Physiological System," as Dr. Broussais (who, by the bye, has no more claim than we have to its formation,) is pleased to call it.

Dr. Broussais is a person of good education and respectable attainments. Many of these he owes to subsequent application, and a conviction that, without them, he could scarcely have hoped to shine amongst his Parisian brethren, who, it is due to truth to say, are among the most learned and scientific physicians in Europe. Having left France when yet young, his opportunities for acquiring knowledge had been but few, and those often neglected from imperious circumstances. Arrived in Italy, Dr. Broussais felt his own deficiency. He knew that he had learned next to nothing at the Ecole de Médécine, which a hundred *bouleversemens* in the early stages of the Revolution had rendered a fruitless institution: he, therefore, guided by his own good sense, applied himself to the study of his profession in the country in which a military appointment had thrown him; and, among other eminent professors whose lectures he there attended, Tommasini, whom we have all since known in London, was the person whose doctrines he mostly cultivated. At that time, what was called the

Brunonian system prevailed throughout the North of Italy: its glaring absurdities were winked at; its essential defects were explained most complacently, but not reformed; and the perpetual contradictions between its tenets and the ultimate results of experience, were got over with the same facility with which a patient was made to quaff a bowl of punch in asthenic diseases. To have dared to set up against so accommodating and so popular a system, would have been next to madness. Yet, this is what Rasori and, almost simultaneously, Tommasini did,* both orally and graphically: the former, in his History of the Petechial Fever of Genoa, and in his admirable Essays on the Action of Digitalis and Tartarized Anatomy on the Animal Economy; and the latter, in a Treatise on Yellow-fever, and several other equally important publications. In the school of these two eminent practitioners has Broussais gathered those fundamental principles on which he has since built a system which he calls his own,—*mutato nomine tantum;* just as the retailer in court trappings, who, having by a lucky chance succeeded in possessing himself of a costly dress at a cheap rate, covers it with tarnished tinsel to increase, as his own taste suggests, the value of it, and vends it, as his own, to the amateur of private theatricals, or for the purpose of a masquerade, or to assist at a coronation.

The present is the second edition of a book which the author published in 1816, with nearly the same title, and which was introduced to the knowledge of the public in this country through various channels, by means of quotations and occasional references. Its purport then, as in the present instance, was to give an account of what were considered by the author to be the most prevailing doctrines in medicine of the day, accompanied by his own animadversions, and a slight glance at the then rising system of the principal physician of the Hospital of Val de Grace.† This latter object Dr. Broussais has so far enlarged upon in the present edition, that, instead of mere suggestions, hints, and allusions, by which alone the reader could collect, in the first edition, the author's ideas of practical medicine, we are now presented with a code of "propositions," constituting the skeleton of his system, with an appropriate name, to give it dignity, and to secure to it an everlasting reputation. The fact is, that Dr. Broussais has grown bolder since certain journalists on the continent have undertaken to sing his praises, upon a mere national principle, that of securing to France the additional honour of another "homme illustre," without condescending to inquire at what other nation's expence the "illustrious author" has secured to himself a certain degree of notoriety.

As we neither have space nor inclination to enter very minutely into the details of the work itself, we shall limit our labours to the giving of a naked outline of the present edition, in order that those of our readers who may admire its construction may have the opportunity of referring to the volumes themselves for more ample information.

* A general view of the principles of Rasori, Tommasini, and of other promoters of the physiological pathology in Italy, was given in the Proemium to the 43d volume of this Journal.

† The medical appointment of Dr. Broussais.

Dr. Broussais lays the foundation of his "Doctrine Physiologique" in a series of "propositions de médécine," amounting to four hundred and sixty-five, which may be considered as so many points of his "creed," to be looked up to, meditated, and followed, by his proselytes "here and every-where." These propositions are classed into so many sections, the first of which is entitled ***Physiology;*** the second, ***Pathology;*** the third, ***Therapeutics;*** the fourth, ***Corollaries.*** As it is upon a better use of physiology that Broussais particularly piques himself, for the essentiality of his system, and its superiority to every other, it may not be amiss to remark, merely *en passant*, that the following propositions, some very *new*, others very *true*, and here and there a gratuitous one, are among the four hundred and sixty-five by which the Broussaian, or physiological, system is constituted. Our extracts must be limited to only a few samples.

*(Gratuitous.)* I. "La vie de l'animal ne s'entretient que par les stimulants exterieurs." This is one of the hundred absurdities of Brown.

*(New.)* XXVI. "Il est un ordre de nerfs situés le long de la colonne vertebrale." That is to say, there are nerves along the vertebral column; a fact with which Mr. Broussais' readers were, perhaps, not aware before.

*(True.)* XXV. "L'embryogénie est l'ouvrage de la chimie vivante: la sensibilité et la contractilité conduisent l'embryon dans l'utérus; la chimie vivante le développe et lui donne sa sensibilité et sa contractilité particulières: la sensibilité et la contractilité de la mère, en opèrent l'expulsion." This is the neatest picture of conception, gestation, and parturition, that we ever remember to have seen drawn up in so few words. The dry aphoristical language employed by the author on this occasion, would lead one to believe that he can easily *demonstrate* what he has advanced in the above proposition. Unfortunately for him, *demonstration* is the only process wanting to prove the truth of his proposition. There are, however, some hundred more such, the truth of which is equally evident, among the four hundred and sixty-five. As for the examples of Dr. Broussais' disposition of wantonly rioting in the pleasures of chimerical inventions, where one would expect nothing but what is tangible and evident (in physiology), they are so numerous that we cannot venture even upon their computation. What do our readers think, for instance, of the following flight of fancy:

XLVII. "Passions, like folly, are the triumph of the viscera, and consequently of instinct, over intellect. Hence they often give rise to folly."—P. xiv.

Into the other sections space will not permit us to enter in detail. Under that of *pathology*, we were struck with Dr. Broussais' ready definition of fever.

CXII. "La fièvre n'est jamais que le résultat d'une irritation du cœur primitive ou sympathique."

With Broussais, every disease proceeds, originally, from gastro-enteritis. Cerebral affections,—essential fevers,—tubercles and cancer—bulimia and chlorosis,—hepatitis and apoplexy,—dropsy, nephritis, scirrhus peritonitis, &c. &c.—all proclaim a common parent, the prolific gastro-enteritis.

Under the head of *therapeutics*, it will be found that "tonics" and "diffusible stimulants" are reckoned amongst the "moyens" for arresting the progress of inflammation. It may be questioned how far, in the case of a late illustrious female, the following directions and opinions of our author would have led to a different result, if acted upon, instead of the usual mode of treating inflammation of the bowels attended with constipation.

CCCXXIX. "La constipation est avantageuse dans les gastro-entérites aigues, parce qu'elle indique que le colon ne participe point à l'inflammation. Elle n'exige autre chose qu'un lavement émollient par jour, quand même elle persisterait, et si la chaleur est considérable, ce lavement doit être donné froid."

Having thus framed a system of medicine by means of various propositions, the majority of which are taken for granted, and are no where discussed and demonstrated, Dr. Broussais proceeds to take a philosophical view of the doctrine of Hippocrates, as well as of those which prevailed subsequent to him, and down to the nosologists of more modern times. Without entering into the correctness of what the author advances with respect to the writings of the Father of Medicine, we venture to assert, that the most important part of Dr. Broussais' observations are taken from an ingenious essay, written several years ago by Rasori, though little known in France, "sul Genio d'Ippocrate." Galen and his sectarians are, of course, not spared. In a separate chapter, the author considers the origin of nosologies, particularly of that of Sauvages, the defects of which he exposes; and slightly touches on the vitalism of the Montpellier school.

We shall spare our readers the trouble of going through the author's *exposé* of the Brunonian system, occupying nearly a hundred pages, and concluding with this very sapient assertion: "Que la classification de Brown en maladies sthéniques et asthéniques, génerales et locales, est purement arbitraire;" as if the medical world were not fully persuaded of the above truism, and had not shown, by their abandonment of a doctrine in itself so absurd, how little they held it in estimation.

Dr. Broussais next enters upon the consideration of what he calls the "Brownisme" of the Italian school. He first shows how readily the Italian physicians adopted the system of the Scotch reformer in the first instance, and next how they subsequently modified it, by various interpolations of their own, so as to create a new doctrine, to which the name of *contra-stimulant* has been given. Dr. Broussais admits that, from so far back as the year 1805, Tommasini had declared that fevers, even after the first days of their invasion, continued to wear the sthenic character, and required for their treatment a corresponding continuation of the antiphlogistic plan;—that the same Professor then taught the very doctrine on which Dr. Broussais has since founded his own, namely, "that the traces of inflammation observed in the viscera after death from fevers, are the cause, and not the effect, of those fevers;" and—that, consequently, Tommasini had completely anticipated Dr. Broussais in those tenets which he afterwards developed in his book on Phlegmasiæ. This concession of our author does not surprise us more than his pertinacity in continuing to call himself the founder of a doctrine which Tommasini has inculcated for the last fifteen years; and to which, as we have already observed, (*mutato nomine tantum*,) Dr. Broussais has merely the credit of having added the knowledge of a physiological fact,—viz. that the seat of inflammation in fevers is either in the mucous or serous membranes. This fact, however, we contend, he has generalized with a facility which actual observation does not warrant us in adopting; for the character, nature, and treatment, of several diseases must be greatly distorted, in order to refer them to an irritated condition of either the serous or mucous tissues of the viscera.

As the confession of Broussais himself on this subject must put an end to all doubts respecting the reality of the claims of the modern Italian school to the foundation of that doctrine which has been, more or less, acted upon in every civilised country in Europe, within the last ten or fifteen years; and must thus effectually silence those expounders of the Broussaian system, who, ignorant of the above historical facts, both here and on the continent, have, either in separate works or in Journals, gone further than Broussais himself, in attributing to the French physician the judicious innovations that have been recently introduced into the practice of physic; we shall be excused if we record it in our author's own words.

"Tommasini publia, dès 1805, que le caractère sthénique des maladies fébriles ne se borne pas aux premiers jours. Au lieu de passer au traitement stimulant pour dissiper la prétendue faiblesse indirecte qui survient, d'après Brown, aussitôt que la prostration musculaire, le rétrécissement du pouls, la couleur terne, les symptômes nerveux, succèdent à la force du pouls à la coloration fleurie des premiers jours, il osa persister dans le traitement antiphlogistique. Bientôt les cures qu'il obtint le convainquirent que les traces de phlegmasies qui se rencontrent dans les cadavres des malades enlevés par les fièvres prolongées, et de toutes les consomptions avec pyrexie, n'étaient point l'effet de la faiblesse indirecte; dès-lors il soutint que la nature sthénique des maladies se conserve la même depuis le premier moment de l'invasion jusqu'au dernier degré de l'épuisement; que toutes les inflammations aiguës, chroniques, évidentes, obscures, sont de cette nature jusqu'à l'entier épuisement des forces, et qu'en un mot *les phlegmasies, dont on rencontre les traces apres la mort, sont toujours la cause et jamais l'effet des fièvres qui ont existé durant la vie.*

"Ce point ayant été éclairci, on s'éleva contre l'assertion de Brown, qui soutient que la majorité est tellement en faveur des maladies asthéniques, qu'il en existe à peine trois sur cent que l'on puisse rapporter à la classe des sthéniques. *La distinctions des inflammations en sthéniques et en asthéniques fut donc regardée comme illusoire et purement spéculative.*

"Il existait, continue Tommasiin dans le discours cité, une autre erreur Brownienne, ayant l'apparence de la vérité. Elle consistait à faire dériver la nature de la maladie, ou la diathèse, de la nature des causes qui l'avaient produite. Cet auteur, étayé par les ouvertures des cadavres et par le succès des antiphlogistiques, soutint que de l'action des causes les plus déprimantes, tels sont le froid et les affections tristes, il résultait une foule de maladies essentiellement et persévéramment d'un caractère sthénique ou de sur-irritation, exigeant imperturbablement un traitement sédatif ; il s'aida, pour établir ces vérités, des vues lumineuses de Gaubius, de Cullen, de Gianini, de Testa, de Monteggia."

The next medical doctrine which Dr. Broussais has undertaken to examine, is that of the German physicians, and those of the North of Europe in general. The two principal physicians, whose works he analyses and comments upon for that purpose, are Joseph Frank and Professor Hildenbrand. The doctrine of the former regarding fevers he considers as vague in the extreme ; and he, moreover, accuses the same author of giving stimulants in fevers. The work upon which this judgment is founded, is the *Praxeos Medicæ Universæ Precepta* of J. Frank, the son of the late venerable P. Frank, whose epitome *De Curandis Hominum Morbis,* possesses considerable merit. Hildenbrand published a work on Typhus, which was translated into French. From the contents of this book, Dr. Broussais judges of the general practice of the German physicians. He admits that, in his views of the fever in question, the German author has considerably approached the "physiological system ;" but he declares that, in the development of those views, and more particularly in his treatment of typhus, the Professor has travelled wide from actual truth. Dr. Broussais, moreover, accuses the German physicians of not knowing the distinction between gastro-enteritis and any other inflammatory disease ; and very shrewdly puts the question, whether they are acquainted with phlegmasia. "Les Allemands connaissent ils les phlegmasies ?" The answer is, "Non." True it is that Schoeffer, a Swiss physician, in a memoir on the Epidemic Disease which prevailed at Ratisbon in 1810, published several years before the appearance of Dr. Broussais' work on Inflammation, had stated that children are very subject to inflammatory complaints, chiefly confined to the mucous membranes of the mouth and cavities of the nose, producing cerebral and pulmonic phlegmasiæ, both chronic and acute ; but, as Schoeffer had unluckily referred the prevalence of the epidemic in question to the atmospherical constitution of the season, Dr. Broussais is unwilling to give him the credit of having anticipated him in the doctrine of membranous inflammation.

Having thus very cavalierly dismissed the consideration of the Italian and German doctrines, our author boldly enters into an examination of the "medecine actuelle de l'Angleterre." How far Mr. Broussais is qualified for the task, may be collected from the circumstances that he is represented to be totally unacquainted with the English language,—that he has never been in England,—and that the only means he has of judging of English practice is through the translations of some few English works, or of some insulated memoirs taken from English periodical publications, and quoted by the French journalists. It is curious, indeed, to see how these circumstances have operated on Dr. Broussais, and have given a colour to his judgment on English medicine. The only authors, whose productions he analyses, and from the nature of whose lucubrations he infers what must be the state of medicine in this country, are a Dr. *Brenan*, of Dublin, (we must give and spell the names as our critic spells them,) a Mr. *I. Thacher*, who happens to be in America, and is an American, (though this seems of no great consequence to Dr. Broussais, in speaking of *English* practice;) *Dr. Hossack*, another American practitioner, whose sins are laid to the account of the English; *Mr. Henning*, who has been foolish enough (says Dr. Broussais,) not to see the connexion between the cutaneous eruption and gastritis in scarlet fever; *Dr. Scudamore*, whose work on Gout Dr. Broussais praises, and whose practice he condemns; the *chirurgien* Newnham,—a great weight, to be sure, in the balance of English practice; a Mr. Bowes, (who is he?) a Mr. Cliston, (who is he?) a Mr. Rogers, (who is he?) Dr. Kinglake, Dr. Burrow, Dr. Sutton, Dr. Park; and, amongst the really eminent, Hunter, Abernethy, and Wilson Philip. Dr. Broussais' authorities are principally the medical Journals; he having in a very few instances, indeed, referred to any of the important works of the last-mentioned authors. As for the Transactions of the Colleges of Physicians of London and Ireland, and of the Medico-Chirurgical Society of London, in which are deposited, we venture to say, more practical facts and useful doctrines than our critic will ever be able to comprehend, Dr. Broussais never hints at them, except in one single instance—that of Dr. W. Philip's paper on Hepatic Phthisis. We must, however, do justice to Dr. Broussais in one point, and that is in the analysis he has given of Hunter's theory of inflammation. To say, however, that all the practitioners in England act upon that theory, is absurd.

Looking at the picture of English practice, as drawn by the great proportion of the artists which DrBroussais has named and selected, and with whose translated works alone he seems to be acquainted, no wonder that he should bring forward, against the English physicians of the present day, a string of accusations like the following: 1. Les medecins Anglais affaiblissent et stimulent dans les maladies aiguës; 2, ils ignorent la cause des gonflemens mesenteriques; 3, ils abusent des purgatifs; 4, ils ne connaissent pas bien les phlegmasies eruptives; 5, ils voient mal les maladies des pays chauds; 6, ils connaissent peu la peritonite chronique; 7, ils stimulent dans le cholera; 8, ils meconnaissent une cephalalgie gastrique; 9, ils visent à l'extraordinaire; 10, ils ont inventé une phthisie dyspeptique; 11, ils meconnaissent les differentes formes de l'irritation; 12, ils sont empiriques.—It is needless to remark, that the defence of the English practitioners from these charges would be a task of no difficulty, were they even founded upon any thing like evidence, or brought forward by a person acquainted with their real practice. As it is, no one will feel inclined to enter the field of discussion with a writer who, besides being totally disqualified to take any part in it, is disingenuous enough, or ignorant enough, not only to confound the reveries of obscure correspondents in periodical Journals, with the superior performances of men of real genius and experience; but also to amalgamate the pretended errors of foreign writers with those of the English, with a view evidently, of increasing the weight of culpability he attaches to the latter in the eyes of the public. Thus, after descanting upon the pretended failures which attend English practice, as collected from the *popular* writings of Messrs. Newnham, *Bowes*, and *Cliston*, the only authors quoted by Dr. Broussais, this critic proceeds to observe, "Ce n'est pas seulement dans leurs echecs que les *medecins Anglais* sont redoubtables. Leurs querisons m'ont souvent fait trembler;" and he then quotes, in support of it, the history of a case taken from the *New-York* Repository!

A worthy contemporary of ours had, on one occasion, taken much pains to develop the "Systeme Physiologique" to the English readers in his Journal; and, being struck with the great truths it contains amidst much dross, without stopping to inquire whether those truths belonged really to the French physician, or to those of another nation, as we have shown in the present article, condescended to extol the system itself, as well as the author, in a very flattering manner. Unfortunately for the value which our contemporary may attach to the praises he might have expected from Broussais in return, his better judgment induced him to add to his eulogium of the French system some very appropriate remarks on the use of certain therapeutical means, which Broussais had either condemned or neglected. This circumstance has drawn upon the reviewer of the "Systeme Physiologique" the wrath of its author, who cannot admit that any thing is wanting to its perfection; and, in the chapter which we have been analysing, he declares, that his "confrère d'Angleterre, bien qu'avec l'intention de se montrer impartial, a cédé a l'influence de la doctrine empirico-Brownienne de son pays et que cette influence l'a exposé, malgré ses bonnes intentions, aux reproches d'inconsequence et de légèraté." But sure we are that the "confrère d'Angleterre" knows fully how to appreciate both the encomiums and the condemnation of our author, of whose impartiality we have already given some few specimens, and of whose modesty the following may be taken as two out of two hundred examples:

"C'est des cours particuliers que je fais que les vérités dont brille la *doctrine physiologique* se sont repandues dans le commerce social et se sont introduites parmi les médecins."*—"Si j'en crois mon préssentiment elle doit avoir prochainement sur la population une influence plus marquée que la découverte de la vaccine."†

Having proceeded thus far in our analysis of Dr. Broussais' two volumes, it becomes almost unnecessary to follow him in his examination of the Spanish and the French Schools. The former is too insignificant, in reality, to deserve much notice; and the latter will find much more powerful defenders amongst the many very illustrious men of whom France can boast, if they should think it adviseable to notice the misrepresentations of one who has equally condemned Barthez, Bordeu, Pinel, Cabanis, et "la médecine Française en général."‡

---

* Preface, p. iv. † *Idem*, p. xii.

‡ A defence of the French school against the attacks of Dr. Broussais has just made its appearance: it is from the pen of M. Authenac, d. m.

*Memoria sopra il Metodo di Estrarre la Pietra dalla Vescica Orinaria per la via dell'Intestino Retto.* Di Andrea Vacca Berlinghieri, Professore di Clinica Chirurgica nell'Imp. E. R. Univ. di Pisa, Cav. dell'Ordine del Merito sotto il Titolo di S. Giuseppe, e Membro di molte illustri Accademie Europee. 8vo. pp. 82. Pisa, 1821.

The objects of this memoir are to render more generally known, in Italy, the author says, the new mode of operating for the stone proposed by Dr. Sanson, (an account of which was given, a short time since, in this Journal;) to render evident the advantages of this mode of operating; and to support the propriety of it, not only by reasoning, but also by some cases of a very important character: and, as facts persuade more than theory, Professor Vaccà thinks that he may hope to be more successful than Dr. Sanson had been in his endeavours to make surgeons in general adopt this, as he considers it, improvement in the art of surgery.

We shall give an abstract of the most remarkable parts of this memoir, (which merits every praise due to a work equally characterized by candour, liberality, and intelligence,) without adducing any considerations of our own on the merits of the practice which Professor Vaccà advocates. The Professor has evidently thought well on this subject, and has presented a very perspicuous view of its relative advantages; whilst the modifications he has effected on the mode of Sanson show, as far as the evidence of six cases extends, that the principal relative disadvantage of it resulted from a circumstance which might, without much difficulty, be obviated. Of the two chief objections to the method of Sanson,—the consequences of the wound of the rectum, which appertain especially to the part itself; and the passage of fecal matter into the bladder, and, hence, a recto-vesical fistula;*—the latter alone, the Professor considers, is of any validity, and it is this which he has obviated by the modification of the operation above alluded to. The method of Professor Vaccà consists in cutting the urethra, the prostate, and the neck of the bladder, and avoiding the *bas-fond* of this viscus. In this operation, the incision of the intestine is at least an inch lower than that of the neck of the bladder, and the edges of the wound of the lower part keep in contact except when the urine is passed, and the parietes of the intestine serve as a valve which opposes the passage of feces into the bladder. The truth of these statements is proved by the six cases in which the operation in question was performed by Professor Vaccà.

The author enters into an examination of the relative merits of the several modes of lithotomy hitherto proposed, the results of which lead him to infer that the recto-vesical operation "seems to unite all the principal advantages, and present the smallest inconveniences."—"It is not necessary," he says, "to recal to the mind of the reader knowledge of minute anatomy, to persuade him that there is no point of the perineum nearer to the bladder than that which corresponds

* This occurred in the cases of Dupuytren, Barbantini, and of Professor Geri, at Turin.

to the anterior part of the sphincter of the anus. Not much ingenuity is requisite to enable us to conceive that, on dividing this point of the sphincter, the parietes of the rectum, the membranous portion of the urethra and the prostate, by an incision which intersects but few soft parts, we shall have procured a very ample space for the entry of the finger, the forceps, and the passage of the stone; because we profit by the natural aperture of the anus, as well as by the cavity of the rectum. The grossest anatomical information is sufficient to lead any person to perceive that an incision which intersects the sphincter of the anus in its anterior part, the membranous portion of the urethra in the median line of its anterior paries, the neck of the bladder, the prostate, and the lower surface of the bladder, in the same line, does not ever encounter any important vessel, or any other part interesting to life. It is clear that, by this method, the stone has to pass between the rami of the ischium, where they are most distant from each other, and where, consequently, they leave ample space for the passage of the largest stone. It is also evident, that the direction and the shortness of the wound render impossible any extravasation of urine, as well as facilitate the egress of any fragments which may have remained in the bladder after the operation. The traject of the wound being shorter than in the other method, the surgeon can penetrate far into the bladder with his finger; ascertain the form, volume, and direction, of the stone; and, according to its situation, easily seize and extract it. In the recto-vesical operation, we avoid the danger of wounding large vessels in the perineum; we may extract large stones as well as in the high operation; whilst this method is devoid of the inconvenience of exposing the peritoneum, and submitting it to the hazard of being wounded by the surgeon, or the other very serious accident of rendering easy the extravasation of urine."

Professor Vaccà thought favourably of the high operation a few years since; but the results of his experience, and further considerations on it, have led him to alter his opinion in this instance. Notwithstanding the presence of a catheter in the bladder, effusion of urine into the cavity of the pelvis took place in the cases in which he performed the high operation, and this in a female as well as in male patients. This he considers the chief relative disadvantage of this operation, though much difficulty in it ensued, in one case, (where the subject was a man twenty-five years of age,) from another cause that had not, we believe, been previously noticed: that is, from violent contraction of the abdominal muscles, after they had been cut, which rendered the wound so thick as not to admit even a finger without extreme difficulty, and obliged the operator to postpone the extraction of the stone to a subsequent period. The same reasons which lead Professor Vaccà to prefer the recto-vesical operation in man, militate, he thinks, in favour of the vagino-vesical operation in woman.

The author has performed the recto-vesical operation in six instances. In four of these cases, (the subjects of which were patients of the ages of 38, 74, 38, and 2, years,) the results were of the most favourable kind: in one (the subject of which was five years old,) a little fistulous communication existed between the rectum and the bladder for nearly a year after the operation. This resulted, the author thinks, from the surgeon into whose care the patient was given, (in the absence of the Professor,) having neglected to touch the edges of the wound of the intestine with argentum nitratum,* (a practice adopted by Professor Vaccà, as soon as inflammation has subsided and suppuration established, and effected throughout the whole traject of the wound that corresponds with the incision of the intestine and the perineum.) In the other case, the patient (a man seventy years old,) died on the fourth day after the operation. Dissection showed inflammation of the peritoneum and of the left kidney. The cellular substance which united the anterior part of the bladder to the pubes was also gorged with blood, and bedewed with puriform serum. The parietes of the bladder, at its upper and anterior surface, were much thickened; there was puriform serum between its muscular and peritoneal tunics: the internal membrane was gangrenous, and presented, at its left lateral portion, some protuberances which contained several very small fragments of stone. It appears that the stone had been adherent to this point. The wound, also, presented a gangrenous appearance. The liver was very voluminous, and occupied a great part of the left hypochondrium.

To the general abstract above given, we shall add the detail of the process of the operation, according to the method of Professor Vaccà, as it is described by the author himself.

The necessary instruments are only a common grooved staff, an ordinary straight bistoury, forceps, and, in some cases, a perfectly straight probe-pointed bistoury.

The patient being placed and secured as for the lateral operation, the staff is passed into the bladder, and then confided to an assistant, who is to hold it firmly in an axis perpendicular to the pubis, without inclining it either to the right or the left, and keep the groove of it pressed against the median line of the urethra, opposite to the raphé. The surgeon then takes the bistoury in his right hand, so that he may cut with it from within outwardly; that is to say, with the cutting edge turned upwards, and the fore-finger and thumb on the point of conjunction of the blade with the handle, so that both of them may be grasped. The fore-finger of the left hand is then oiled, and one of the sides of the bistoury pressed very firmly against its palmar surface, so that its cutting edge may be a little below the upper surface of the finger, and form with it, as it were, one body, which may be passed into the rectum without wounding the patient. The finger and the bistoury are then introduced through the anus, with the dorsal surface of the finger opposite to the sacrum, and advanced to the distance of ten or twelve lines from the verge of the anus; the finger is to be pressed

* This patient was received again into the hospital, after the lapse of the period above-mentioned, and the caustic applied. When this memoir was published, (April the 24th, 1821,) the wound "was in such a state as to lead the author to hope that it would be completely cured in a very short time;" and then only from four to six drops of urine passed by the fistula, whilst several ounces were evacuated by the urethra.

against the posterior, or sacral, surface of the rectum, in order that a subsequent necessary change in the position of the instrument may be effected: this change is made by means of the right hand, and consists in applying the back of the instrument to the palmar surface of the finger, and its cutting edge to the anterior part of the intestine, exactly in a line corresponding with the raphé of the perineum. The finger, which had been introduced to facilitate the change of the position of the bistoury, is then pressed forward against the instrument, the cutting edge and point of which, being fixed in the anterior part of the intestine, whilst the right hand, drawing the knife from the intestine, will co-operate in making an incision of the anterior paries of the intestine, the cellular texture situate between this and the urethra, and the external sphincter of the anus; beyond which sphincter the incision should not extend above the distance of eight or nine lines in the perineum. On this being effected, (which is done in an instant,) the operator removes the left fore-finger from the bistoury, alters the direction of this finger, turning to the left its dorsal surface, and at the same time, by a light movement, changes the position of the instrument; so that its cutting edge, which had been turned upwards and towards the operator, is placed in an exactly opposite direction. The operator now passes the end of his left fore-finger into the wound, exactly on a parallel with the incision in the sphincter, and seeks, with the edge of the nail (which should always be long when this operation is performed), the groove of the sound through the parietes of the urethra. On the groove being found, the bistoury is carried, with its back upwards, to just above the nail of the left fore-finger, and the urethra is cut by the instrument; which, with the nail of the fore-finger, enters the groove of the staff still held by the assistant in the situation before described. The instrument is now thrust forward, by the right hand, into the bladder, and the neck of this viscus divided to a greater or less extent, according to the notions that may have been formed of the bulk and figure of the stone. As we are very apt to err in this point, it is adviseable that the wound in the neck of the bladder and prostate should be rather small, as it can very easily be enlarged subsequently, should this prove necessary. On this incision being completed, the finger is passed (having the staff as a guide,) into the bladder. We can then judge, by means of this finger, of the size of the wound, as well as of the size and form of the stone, and, according to the indications of this examination, be induced to enlarge the incision, or let it remain of its present dimensions. If it be thought proper to extend it, the bistoury already used may serve for this purpose; but, as the point of the ordinary bistoury might embarrass the surgeon and expose him to the danger of wounding his finger, and perhaps the bladder, unless it were very expertly managed, it is better to employ here the straight probe-pointed bistoury, by which both these accidents may be avoided. The forceps are passed along the finger into the bladder: all the guides and gorgets invented for conducting the forceps are perfectly useless instruments in this, as well as (the Professor says,) in all the other methods of extracting the stone. The wound after the operation is treated in the ordinary manner: the

Professor thinks that the use of sutures in the wound of the sphincter of the anus, proposed by Dr. Sanson, is unnecessary and injurious. The only surgical measure generally requisite is the application of caustic to the edges of the wound, in the manner already described. "This practice," says the author, "serves wonderfully to accelerate cicatrization; and the only one of my patients that was not promptly cured, was that in whose case the argentum nitratum was not used from an early period, in consequence of my being obliged to intermit my attendance at the hospital, when the young surgeon interested with the care of this patient did not carefully employ a remedy which appeared to him to be too hazardous."

