# Errata in Last Number

**Published:** 1821-11

**Authors:** 


					V-i ,'.ne '?ln 'he top, for aware iciul acquainted.
?   ;?>' ? i'?c 10 from the bottom, lor thick read strict.
-131, line 1) from the top, lor interested ic:ul intrusted.

				

